# What Is the Most Sensitive Test to Identify Fatigue through the Analysis of Neuromuscular Status in Male Elite Futsal Players?

**DOI:** 10.3390/s22207702

**Published:** 2022-10-11

**Authors:** Adrián Moreno-Villanueva, Markel Rico-González, Carlos D. Gómez-Carmona, Miguel Á. Gómez-Ruano, Nuno Silva, José Pino-Ortega

**Affiliations:** 1 Faculty of Sports Sciences, University of Murcia, 30720 San Javier, Spain; 2 BIOVETMED & SPORTSCI Research Group, Department of Physical Activity and Sport, Faculty of Sport Sciences, University of Murcia, 30720 San Javier, Spain; 3 Department of Didactics of Musical, Plastic and Corporal Expression, Faculty of Education, University of the Basque Country (UPV/EHU), 48940 Leioa, Spain; 4 Faculty of Physical Activity and Sport Sciences, Technical University of Madrid, 28040 Madrid, Spain; 5 Research Center in Sports Sciences, Health Sciences and Human Development (CIDESD), University of Maia (ISMAI), 4475-690 Maia, Portugal

**Keywords:** neuromuscular fatigue, team sports, training load, back squat, countermovement jump

## Abstract

The present study aimed to determine which of the neuromuscular status (NMS) monitoring tests (1: Counter-movement jump, CMJ; 2: back squat with additional load) is the most sensitive and effective for evaluating the state of fatigue in futsal players during the preseason. Seventeen professional futsal players were recruited for this study (age: 23.07 ± 6.76 years; height: 1.75 ± 0.06 m; body mass: 75.47 ± 7.47 kg; playing experience in elite: 5.38 ± 2.03 years). All of them were evaluated during the preseason phase in two tests (CMJ and back squat with additional load) before and after each training session (pre- vs. post-test). A jump platform was used to extract jump height during CMJ, while a linear position transducer was used to extract mean velocity (MV) and mean propulsive velocity (MPV) during the back squat exercise. Significant differences were obtained for intra-subject analysis for MV and MPV in loaded back squat exercise (*p* < 0.001), finding lower values during the post-test. In conclusion, the monitoring of NMS through the back squat provides greater sensitivity and objectivity in comparison with CMJ, due to a more direct neuromuscular extrapolation to the physical demands of futsal.

## 1. Introduction

It is widely accepted in the sports science area that the preparatory training periods, especially the preseason, constitute the main period for improving the performance of athletes in team sports such as futsal [1,2]. However, the congested fixtures in team sports (e.g., soccer, basketball, and futsal) have reduced the preseason duration, forcing the coaching staff to concentrate a high workload in a concise time (e.g., 3–4 weeks) [3,4,5]. For this reason, the risk of players suffering episodes of acute or chronic fatigue during the preseason increases considerably, which can lead to non-functional overreaching, injuries or illness [3]. Therefore, the monitoring of the acute state of fatigue in futsal players during the preseason is a topic of great interest today, and the understanding of “state of fatigue” as a transient and sharp decrease in sports performance, including the decreased performance immediately after a training session and/or competition [6].

Futsal is characterized as a high-intensity intermittent sport that imposes high physical, technical, tactical and psychological demands on the players, with average intensity values of ≥85% maximal heart rate and ~80% maximum oxygen uptake [VO2max] [7,8,9]. The game takes place on a 40 × 20 m field during two 20-min parts of stopped time (separated by a 10-min break), played by 5 players (4 field players and one goalkeeper) on each team, plus a maximum of 9 substitute players per team, with an unlimited number of substitutions [10]. Currently, little is known about the neuromuscular demands at the elite futsal level. Although, there is a clear consensus that players need to develop and improve parameters such as maximum speed, power and lower body strength, as well as aerobic capacity [7].

Player fatigue can be monitored using biochemical (e.g., creatine kinase, lactate dehydrogenase, oxidative stress markers) [11,12], physiological (e.g., heart rate variability, heart rate recovery index) [13,14], psychological (e.g., rating of perceived exertion, athlete self-report measures, profile of mood states, total recovery scale) [4,15,16] and physical performance variables [11,17,18,19]. Although physical performance can be evaluated through multiple performance tests (Yo-Yo test, repeated sprint ability, 5 m and 20 m sprint, *t*-test) [2,20], the evaluation of the neuromuscular status (NMS) in athletes offers objective and useful quantifiable results [21] without the need to invest too much time for it. In this sense, the height obtained in the countermovement jump has been widely used to monitor the NMS of team sports athletes due to its associated validity [22] and reliability [23], and its little need for time to carry it out. During countermovement jump, an explosive action of the lower limb is produced that is typical of sports modalities with opposition [24]. However, countermovement jump testing requires the application of protocol requirements that consume valuable time for training, since it is not integrated into any specific training program [25]. Other studies have analyzed the NMS through other tests, such as the ballistic hip thrust test [26], or through treadmill tests such as the maximal anaerobic running test (MART), a progressive intermittent test that culminates with volitional fatigue [27]. Therefore, the search for an alternative method of monitoring the NMS that can be integrated into a training session (e.g., within specific strength training sessions) may result in greater optimization of it, both in terms of time and the quality of the results obtained.

Previous studies reported a strong negative correlation between execution velocity and relative loads based on one repetition maximum (1RM) [28,29]. In addition, by knowing the load-velocity profile of an athlete, it is possible to determine velocity loss associated with a given load after training or competitive events [17]. Therefore, the bar velocity loss during resistance exercise can be used in combination with countermovement jump variables to evaluate changes in NMS after fatiguing episodes [30,31]. Strong positive correlations have been reported between changes in countermovement jump height and bar mean propulsive velocity during the back squat (*r* = 0.93) at relative intensities of 60–90% of 1RM after resistance exercises [30,32]. However, it is not known which test provides more sensitive information regarding fatigue accumulation during team sports training sessions, or if they are comparable for detecting performance loss.

The countermovement jump has been used extensively to quantify neuromuscular fatigue in team sports [17,22,24,33]. However, as far as we know, bar velocity in the squat exercise to quantify neuromuscular fatigue has been performed only in official rugby matches [17]. If the velocity of the barbell in the loaded back squat exercise proves to be sensitive enough to identify states of acute fatigue based on changes in the NMS, it would represent an advance in the economy of training time, since such an exercise could be integrated into strength training programs while monitoring the state of fatigue of athletes. Therefore, the aim of the present study was to determine which of the NMS monitoring tests (countermovement jump vs. back squat with additional load) is the most sensitive to identify states of acute fatigue based on changes in physical performance of futsal players in training sessions during the preseason. We hypothesized that both tests would present sensitivity when quantifying the NMS, but that the back squat test with additional load would have greater sensitivity than the countermovement jump test, mainly due to the low intra- and inter-subject variability in the execution of the back squat technique.

## 2. Materials and Methods

### 2.1. Participants

17 male players (including 3 goalkeepers) from a First Division Portuguese futsal club, all of them professional players, were recruited voluntarily to participate in this study (age: 23.07 ± 4.53 years; height: 1.75 ± 0.06 m; body mass: 75.47 ± 7.47 kg; playing experience in elite (number of years played in first divisions): 5.38 ± 2.03 years). The exclusion criterion was the existence of any recent injury that required medical attention. The study was approved by the local Ethics Committee (ID: 3495/2021) and followed the ethical recommendations for studies in humans established by the Declaration of Helsinki (2013).

### 2.2. Instruments

Two instruments were employed to provide data about the NMS of futsal players during the assessments:

- *Chronojump Boscosystem (Barcelona, Spain)*: The system is composed of a chronometric device that registers the timing changes in the status of the detection device, called Chronopic. The data are collected through its “Chronojump” management software, through a Computer with a Windows 10 operating system. This system was configured with a threshold of 50 ms. This instrument was used during the countermovement jump to directly extract the time of flight and to then calculate the jump height.
- *Encoder ADR*: This device consists of a linear position transducer technology that transfers the concentric velocity data instantly via Bluetooth to a portable device with customized software (version 5.2). It has a sampling frequency of 1000 Hz, with a calculation error of ±2.5 mm of displacement and a spring tension of 150 g (data provided by the company). This instrument was used during the back squat with additional load to extract two variables: (1) mean velocity (MV) which is calculated as the value from the start of the concentric phase until the bar reaches the maximum height; and (2) mean propulsive velocity (MPV) which is calculated as the value from the start of the concentric phase until the acceleration of the bar is lower than gravity (−9.81 m·s^2^).

### 2.3. Measurements and Experimental Procedures

The study was carried out before (pre-test) and after (post-test) each training session (*n* = 16) during the preseason (from 16 August to 12 September 2021) involving a Portuguese professional futsal team. The training sessions were carried out with a frequency of 4–5 days a week for 3 weeks, with a duration of each session of 98 ± 7 min. The participants were exposed to an initial week (from 16–22 August) of familiarization with the tests that were carried out during the research protocol, to guarantee a correct technique and consistency in the execution.

An initial submaximal test with progressive loads (series of 5 repetitions, with an initial load of 20 kg and progressive loads of +5 kg between series, with 5 min of rest between series, up to a maximum of 4 attempts/series) was developed to create individualized force-velocity profiles for each participant after the familiarization process. This protocol establishes the load that corresponds to an MPV of 0.9-to-1.1 m/s during the back squat, where the maximum power of execution is reached in each player [34,35]. Subsequently, the players were divided into two groups with a constant load of 30 kg or 40 kg and assigned to one or the other group depending on the most optimal adaptation (e.g., the load that best approximated 0.9 m/s, to try to prevent possible adaptations throughout the preseason from increasing the MPV values above 1.1 m/s) to the load range previously exposed. Furthermore, these loads were subsequently used to monitor the NMS throughout the entire intervention.

A standardized 12-min warm-up was performed before any test of the intervention protocol, which consisted of joint mobility exercises, short and fast displacements in multiple planes, 4 submaximal countermovement jumps, and 12 squats with an unloaded bar. After the warm-up, players performed countermovement jump and squat tests, in this order, so as not to cause interference between tests. Each player carried out each test with the futsal boots with which he usually trained and played. A 3-min break between exercises was respected during assessments. The protocol followed is detailed below:

- *Countermovement Jump:* Participants performed the countermovement jump (*n* = 3) from an upright position, using a self-selected depth, and were instructed to jump as high as possible while keeping their hands on their hips throughout the jump. Ten s of standing passive rest were given between each attempt [30,36]. The height of the countermovement jump has been suggested to be sensitive to changes in NMS in team sport athletes [24,36]. Therefore, the jump height variable was selected to analyze pre-training and post-training countermovement jump performance.
- *Back squat with additional load:* The depth of each repetition of the back squat (*n* = 3) was previously determined and standardized (90°, femurs approximately parallel to the ground) during the quasi-isometric portion of the exercise. Once the starting position was established, the participants had to perform the concentric phase as quickly as possible until they reached full extension of the hip and knee without jumping [35]. During each repetition, the bar remained in constant contact with the upper surface of the trapezius muscle [28]. Throughout the study, foot placement and squat depth were determined and replicated throughout all test procedures for each participant placing floor markings and bands to provide references for maintaining the consistency of motion range [37].

The MV and MPV of the barbell were recorded during each squat using a linear position transducer (ADR encoder; Toledo, Spain), which was fixed to the bar within the collar and the ground directly below the bar [37]. For the development of the load-velocity profiles, standard linear regressions were calculated using the fastest MPV repetition of each series with submaximal loads. The load-velocity profile is highly reliable when using the back squat with loads between 20–90% of 1RM (ICC > 0.8; CV < 7%) [38]. The section of the regression line was determined at an MPV between 0.90–1.1 m/s and the corresponding load was calculated with a precision of 2.5 kg. Once the individualized force-velocity profiles had been performed, each athlete performed two repetitions with an additional load corresponding to 60% of 1RM (0.90-to-1.10 m/s), with a 10 s rest between repetitions.

This protocol was repeated in pre-training and post-training assessments during the preseason, to evaluate performance decreases derived from a state of acute fatigue, caused by loads of the training session. If changes in performance are not detected in any specific player, it could be interpreted that the training loads were not sufficient to generate a state of non-functional overload on the athlete’s physical condition.

### 2.4. Statistical Analysis

Before analyzing the study’s variables, data normality assumptions were tested using the Shapiro–Wilk test (*p* > 0.05). Descriptive statistics were presented as mean and standard deviation (M ± SD). In addition, the delta values (before–after) and the minimum detectable change were calculated to estimate the significant value of change (before vs. after) for each test. A one-way ANOVA for repeated measures was realized to compare the differences between pre- and post-measures (intra-subject measures). The Bonferroni post hoc test was used to analyze the pairwise comparisons, and the effect size (ES) was estimated using the partial eta square for the ANOVA results and Cohen’s d for intra-subject comparisons. The ranges of interpretation for both ES are as follows: (i) partial eta square: small = 0.01; medium = 0.06; and large = 0.14; and (ii) Cohen’s d: trivial < 0.2; small = 0.2–0.6; moderate = 0.6–1.2; large = 1.2–2.0; very large > 2.0 [39].

Then, the temporal series of players’ performance was analyzed using the autocorrelation function to check the variability of measures (accounting for 1-lag = 1 training session). The autocorrelation function provides positive or negative values indicating higher or lower variability (persistency) of measures for each test during pre- and post-test. The cross-correlation function was performed for each test to control for correlated or non-correlated measures between pre- and post-test. Positive or negative values allow the identification of an increased or decreased coordination between measures. In order to check the magnitude of correlations the following range of *r* values was considered for: trivial < 0.1; small = 0.1–0.3; moderate = 0.3–0.5; large = 0.5–0.7; very large = 0.7–0.9; nearly perfect > 0.9 [40]. The significance level was established at *p* < 0.05. Data analysis was performed with the Statistical Package for the Social Sciences (SPSS Statistics, version 24, IBM Corporation, Armonk, NY, USA).

## 3. Results

The post hoc analysis indicated significant differences back squat with additional load variables (MV: *p* < 0.001; ES = 0.552; MPV: *p* < 0.001; ES = 0.628) between pre- and post-intra-subject measures (see Table 1). 

The descriptive statistics (mean ± standard deviation) for the variables studied, and the intra-subject comparison results through Cohen’s d are presented in Table 2 for all players. The analysis showed statistically significant differences for intra-subject effects for MV and MPV in loaded back squat exercise (*p* < 0.001) with lower values during the post-measures. The analysis of the temporal series for each player and all players using the autocorrelation function of pre- and post-measures are available in Table 3. The autocorrelation function results showed non-persistency (non-significant; *r* < 0.5) for pre- and post-measures of countermovement jump, MV and MPV. In addition, the cross-correlation function results between pre and post measures showed a large value for countermovement jump (*r* = 0.68), a very large value for MV (*r* = 0.89) and a nearly perfect value for MPV (*r* = 0.91).

## 4. Discussion

No previous study has investigated whether the evaluation of MV and MPV during the propulsion phase of a back squat with additional load is more sensitive for monitoring fatigue status through the NMS during the preseason, compared to the countermovement jump test commonly used for this aspect. Therefore, the study aimed to determine which of the NMS monitoring tests is more sensitive and effective for evaluating the state of fatigue in futsal players during the preseason based on pre- and post-training performance in each session. The conclusions drawn almost corroborated our initial hypothesis. The results of the present investigation suggested that the variables of the concentric phase MV and MPV of the back squat with additional load were more sensitive and reliable to changes in the NMS and the state of fatigue compared with the jump height measured in the countermovement jump test. However, the differences in sensitivity between both tests do not seem to be due exclusively to the standardization of technical execution, but also due to the request of more specific muscles (quadriceps and gastrocnemius) in the futsal modality by the back squat test with an additional load.

In futsal, neuromuscular function decreases in the preseason phase due to muscle damage caused by the accumulation of training loads during specific strength sessions or during game-based situations where repeated eccentric loads such as decelerations and changes of direction are performed [7,41]. These overload episodes are usually concentrated in specific muscles in response to the demands imposed by the sport such as quadriceps and gastrocnemius which play a fundamental role in running decelerations and changes of direction [42]. These findings are consistent with the significant decrease in the maximum voluntary knee extensor contraction and the torque development rate found immediately after a soccer game in semi-professional athletes [43] and after a rugby match in amateur athletes [44]. Therefore, it is expected in futsal that the workload demands provided from accelerations and decelerations will be considerably higher due to a reduced area per player compared with soccer and rugby as well as an unlimited number of substitutions [7,45]. In the present study, the most accurate and stable values to identify episodes of fatigue through the NMS evaluation proved to be those from the additional load back squat (see Table 1 and Table 2). This statement corroborates the conclusions obtained from previous studies, which suggested that the back squat requests muscle activation in a more localized and specific way [46,47], coinciding with the most demanded muscle groups in futsal, such as the quadriceps and the gastrocnemius [42].

Considering variability, the back squat technique was standardized with identical performance criteria (knee flexion and foot placement) among the participants to reduce the inter-individual variability of results [35,48]. In addition, the biomechanical nature of the back squat makes it possible to be focused on the quadriceps and gastrocnemius muscles [46,47]. However, the countermovement jump execution technique was more flexible, allowing participants a self-selected depth, as the vast majority of previous investigations have shown [21,25]. In this way, the athletes could have adapted the depth of the knee flexion in the countermovement jump execution to engage posterior chain musculature through greater participation of the hip extensors and optimize the performance of the countermovement jump test, limiting the recruitment of fatigued muscles (i.e., quadriceps and gastrocnemius) [49,50]. These factors (e.g., the non-strict standardization of technical execution and the solicitation of large muscle groups not only specific to futsal) have been able to explain the higher fluctuation and lower precision in the values derived from the countermovement jump test compared to the back squat test. Therefore, the back squat test may have a more direct extrapolation to the principal muscles involved in futsal than the countermovement jump test.

Together with the biomechanics of movement, the resistance to overcome in the different performance tests can play a fundamental role in the consistency of the results [51,52]. In the countermovement jump test, players only need to overcome the resistance of gravity. This factor, together with the interindividual variability in the technical execution of the movement, implies obtaining fluctuating jump height results (Cross-correlation function = 0.68; *r* = 0.5). On the other hand, in the back squat test, the players have to overcome the resistance of gravity plus an additional load, established individually according to the previously personalized force-velocity profile. In the back squat, two aspects produced greater demands on the muscles of the lower body in comparison with the countermovement jump test [51,52]: (1) the higher force required to overcome an additional load; and (2) the higher range of movement imposed which produced an increase in stretching and longer concentric duration. These results are similar to those reported by Callaghan et al. [17], which suggested that the additional load in the back squat may impose higher requirements on the muscles of the lower body in sub-elite level rugby players. Considering this previous factor and the direct recruitment of the most fatigued muscles during a futsal match [7,42,46], the monitoring of the NMS through the back squat provides greater sensitivity and objectivity than the countermovement jump.

Although this study offers novel information on the analysis of MV and MPV for the assessment of the NMS and the identification of episodes of acute fatigue in elite-level futsal players, different limitations of the study should be mentioned. First, although it has been shown that the use of a load corresponding to 60% of 1RM can be used to monitor the NMS through the MV and MPV in the back squat with additional load through a linear position transducer [30,32], this device does not detect changes in the vertical components of the maximum power as using a force platform [21,53]. Second, the findings of this research are framed in a very specific scenario such as the preseason and with a limited group of elite futsal athletes. Therefore, future research should analyze following the protocol of evaluations provided in this research that the results can be extrapolated to other team sports, levels of athletes (e.g., youth, and semiprofessional), as well as to other season periods. Finally, although external performance data were obtained during the training sessions through electronic tracking devices, the countermovement jump and back squat performance data did not correlate with these types of variables. This factor could have been crucial in determining more objectively and rigorously if the data obtained in the present study are mainly due to the physical demands of the training, or to an accumulation of fatigue throughout the week.

## 5. Conclusions

This is the first study to compare the sensitivity and efficacy of the back squat with additional load and countermovement jump to monitor the NMS and evaluate the fatigue status of professional futsal players during the entire preseason period. Our findings suggest that: (1) both tests (countermovement jump and back squat) present sensitivity to detect NMS; and (2) the back squat with additional load test (both MV and MPV) has a higher sensitivity for monitoring NMS and the state of fatigue in futsal players. These results may be due to: (1) the greater capacity of the back squat to recruit fundamental muscle groups for futsal practice (quadriceps and gastrocnemius) in a more specific and isolated way; and (2) the greater demand imposed by the additional load to overcome and the range of movement. The findings of the present investigation could help strength and conditioning coaches to monitor NMS and identify episodes of acute and chronic fatigue in athletes validly and reliably through a non-invasive and excessively fatiguing procedure, through easily transportable linear position transducer devices that are cheaper than force platforms.

## Figures and Tables

**Table 1:** Results of the repeated measures ANOVA for intra-subjects factor (pre vs. post).

	SS	DF	MS	*F*	*p*	ES
**CMJ**	9.01	1	9.005	1495	0.223	0.007
**MV**	0.32	1	0.320	305,689	<0.001 *	0.552
**MPV**	0.49	1	0.494	418,758	<0.001 *	0.628

**Note**. SS: Sum of Squares; DF: degrees of freedom; MS: mean squared; ES: effect size. * *p* < 0.001.

**Table 2:** Descriptive statistics (mean and standard deviation) for countermovement jump, mean velocity and mean propulsive velocity variables during pre- and post-measures.

	Pre		Post		Delta		MDC	Cohen’s d
	M	SD	M	SD	M	SD		
**CMJ** (cm)	42.05	4.40	41.73	4.94	−1.25	10.87	6.52	0.246 (small)
**MV** (m/s)	1.08	0.06	1.02	0.06	0.06	0.14	0.18	1.41 (large)
**MPV** (m/s)	1.09	0.06	1.02	0.07	0.07	0.14	0.13	1.628 (large)

**Note.** CMJ: Countermovement jump; cm: centimeters; Delta: Mean differences between pre- and post-measures; MDC: Minimum detectable change; MPV: Mean propulsive velocity; m/s: meters per second; MV: Mean velocity.

**Table 3:** Results of the temporal series for each and all players using the autocorrelation function for each variable and cross correlation function relating to pre- and post-measures.

Subject	CMJ (cm)			MV (m/s)			MPV (m/s)		
	PRE	POST	CCF	PRE	POST	CCF	PRE	POST	CCF
**1**	0.07	0.04	0.38	0.07	0.09	0.65 *	−0.04	0.01	0.84 †
**2**	−0.01	−0.24	0.59 *	−0.23	0.01	0.69 *	−0.09	0.05	0.75 †
**3**	0.04	−0.13	0.53 *	0.27	−0.01	0.86 †	0.20	−0.20	0.67 *
**4**	0.17	0.06	0.82 †	0.10	−0.04	0.93 ‡	0.09	0.10	0.95 ‡
**5**	0.19	−0.02	0.60 *	−0.09	−0.20	0.83 †	−0.03	−0.24	0.65 *
**6**	0.41	−0.02	0.53 *	−0.05	−0.32	0.76 †	−0.11	−0.14	0.80 †
**7**	−0.15	0.29	0.45	0.25	−0.09	0.67 *	0.16	0.10	0.87 †
**8**	0.03	0.01	0.94 ‡	0.15	0.08	0.77 †	0.34	0.02	0.67 *
**9**	0.39	−0.06	0.42	0.32	−0.11	0.93 ‡	0.30	0.03	0.94 ‡
**10**	0.21	0.10	0.72 †	−0.06	−0.09	0.58 *	−0.07	−0.08	0.59 *
**11**	0.29	−0.06	0.57 *	0.19	−0.31	0.75 †	0.32	0.00	0.76 †
**12**	0.43	0.47	0.65 *	−0.05	−0.06	0.91 ‡	0.03	0.01	0.89 †
**13**	−0.23	0.04	0.62 *	0.05	0.06	0.54 *	−0.15	−0.39	0.38
**14**	0.37	0.32	−0.21	0.26	0.32	0.85 †	0.09	0.14	0.82 †
**15**	0.13	−0.20	0.55 *	0.34	0.08	0.61 *	0.14	−0.02	0.67 *
**16**	0.09	0.03	0.37	−0.02	−0.08	0.16	0.00	0.04	0.66 *
**17**	0.44	0.15	0.91 ‡	0.08	0.07	0.91 ‡	−0.08	0.07	0.84 †
**All**	0.11	0.05	0.68 *	0.23	0.15	0.89 †	0.08	0.05	0.91 ‡

**Note.** CCF: Cross correlation function; CMJ: Countermovement jump; cm: centimeters; MPV: Mean propulsive velocity; m/s: meters per second; MV: Mean velocity. **Interpretation of *r* values.** * large (0.5). † very large (0.7); ‡ nearly perfect (0.9).
